# Laser Powder Bed Fusion Parameters Optimization for Enhanced Mechanical Properties of EOS Co-Cr Dental Alloy

**DOI:** 10.3390/ma17204993

**Published:** 2024-10-12

**Authors:** Dalibor Viderščak, Zdravko Schauperl, Biserka Runje, Sanja Šolić, Amir Ćatić, Matjaž Godec, Irena Paulin, Črtomir Donik

**Affiliations:** 1 Faculty of Mechanical Engineering and Naval Architecture, University of Zagreb, Ivana Lučića 5, 10000 Zagreb, Croatia; zdravko.schauperl@fsb.unizg.hr (Z.S.); biserka.runje@fsb.unizg.hr (B.R.); 2 Department of Mechanical Engineering, University North, Jurja Križanića 31b, 42000 Varaždin, Croatia; ssolic@unin.hr; 3 School of Dental Medicine, University of Zagreb, Gundulićeva 5, 10000 Zagreb, Croatia; catic@sfzg.hr; 4 Institute of Metals and Technology, Lepi pot 11, 1000 Ljubljana, Slovenia; matjaz.godec@imt.si (M.G.); irena.paulin@imt.si (I.P.); crtomir.donik@imt.si (Č.D.)

**Keywords:** LPBF, production parameters, Co-Cr dental alloy, CCD, mechanical properties, ANOVA

## Abstract

This research explores how variations in laser powder bed fusion (LPBF) parameters—laser power (*P*), scanning speed (*v*), and base plate preheating temperature (*ϑ*_p_)—affect the mechanical properties of the EOS Co-Cr SP2 dental alloy. A central composite design (CCD) was used to optimize the process parameters. Mechanical testing focused on crucial properties for dental applications, including yield strength (*Rp*_0.2_), elongation (*ε*), toughness (*KV*_a_), and flexural strength (*R*_ms_). Microstructural analysis was conducted using light and electron microscopy, while XRD identified microstructural phases. Statistical analysis (ANOVA, Scheffé post hoc test, α = 0.05) revealed significant effects of *P*, *v*, and *ϑ*_p_ on the mechanical properties. Response surface models (RSMs) were developed, and optimal parameters were determined to achieve maximum toughness and flexural strength. Maximum values were obtained with laser power above 205 W and base plate preheating at 310 °C. The mathematical model predicted toughness values with less than 5% deviation from experimental results, indicating high accuracy.

## 1. Introduction

Over the past years, Co-Cr-based alloys have been widely used in biomedicine, particularly for creating orthopedic implants like hip and knee replacements [1]. In dentistry, these alloys are commonly used for fabricating fixed prosthetic restorations, for example, crowns and bridges [2,3]. The demand for Co-Cr-based alloys is rising, with market projections estimating it will reach $2.6 billion by 2030 [1]. This growth is stimulated by increased investment from different branches of industry, not only due to their importance in medical and dental fields but also because of their applications in advanced industries, turbines, and components of aircraft engines [1,4].

Co-Cr-based alloys offer optimal mechanical properties essential for dental use: yield strength, elongation, flexural strength, and toughness, with high hardness providing excellent wear resistance [1,5,6]. They are also corrosion-resistant and biocompatible due to forming a protective Cr_2_O_3_ oxide layer [7,8]. The mechanical properties of these alloys depend on their microstructure, which is influenced by their chemical composition and manufacturing processes [8]. Microstructure consists of a ductile *γ* phase (FCC) and an *ε* phase (HCP), which influences wear resistance [1,9]. The ratio of these phases, along with carbides, intermetallic compounds, and nitrides, determines overall properties [5,10]. Besides chromium (up to 30 wt%), Co-Cr alloys contain molybdenum and tungsten (around 5 wt% each), with smaller amounts of elements like manganese, silicon, iron, and carbon (<1 wt%) [4,9]. Carbon plays a key role in stabilizing the γ_FCC_ phase, increasing mechanical strength while reducing elongation as its content rises [7,10]. Through the formation of carbides (Cr_23_C_6_, Cr_17_Co_4_Mo_2_C_6_, M_6_C, M_7_C_3_), Cr improves resistance to wear, corrosion, and oxidation and contributes to an increase in hardness [11]. Intermetallic phase Co_3_Mo affects the strengthening at high temperatures [12,13]. Cr and Mo form substitutional crystals mixed with Co [14,15]. W or Mo (or a combination) is added to Co-Cr alloys to achieve a fine-grained structure and improve mechanical properties [11]. This effect is reduced when W or Mo forms intermetallic compounds rather than being uniformly distributed within the Co matrix [16].

To fabricate Co-Cr-based alloys, machines for additive manufacturing using the LPBF process, where metal powders already proven in dental applications, are used [17,18]. In addition to the Co-Cr-based alloys, the exploitation properties and dimensional accuracy of dental structures fabricated in this way significantly depend on the fabrication parameters of the LPBF process and their interaction [19,20,21].

The LPBF process for dental Co-Cr alloys is influenced by key parameters: laser power *P* (W), scanning speed *v* (mm/s), layer thickness *t* (μm), and hatch distance *h* (μm) [22]. These parameters have a significant impact on the microstructure, mechanical properties, and surface quality of the alloys [23]. Higher laser power results in better fusion of powder particles, creating denser structures with fewer pores [24]. However, excessive laser power can cause overheating, leading to undesirable grain growth and reduced mechanical properties [25]. Optimal laser power ensures fine-grain microstructures and enhances properties, hardness, and wear resistance [26]. High values of scanning speeds reduce the interaction between the laser beam and material, which can lead to lower energy input and incomplete melting, resulting in increased porosity and lower density [27]. On the other hand, lower values of scanning speeds can improve fusion but may cause thermal distortion [28]. Balancing scanning speed is essential for achieving dense alloys with good mechanical properties [29]. Thicker layers reduce build time but can lead to lower resolution and less uniform mechanical properties [20]. Thinner layers improve detail and surface finish, contributing to better mechanical properties, but they increase processing time [20,30]. Optimizing layer thickness ensures a balance between build efficiency and mechanical properties [31]. The hatch spacing affects the overall density and microstructure [28,32]. A lower hatch distance results in overlapping tracks, leading to highly dense parts. With the higher hatch distance, unmolten powder may remain, increasing porosity and lower mechanical properties [28]. Base plate preheating minimizes the temperature gradient between the build layer and the substrate (a more controlled solidification process), which reduces the thermal stresses that occur during rapid cooling and solidification [1,33]. Preheating influences finer grain, phase distribution (control the formation of undesirable phases), and uniform microstructure [34]. Preheating reduces the formation of pores and voids by providing higher powder fusion during the laser melting [35]. This leads to denser, higher-quality parts with improved mechanical properties [34]. In summary, base plate preheating in the LPBF process results in improved mechanical properties, reduced residual stresses, higher ductility, and fine grain microstructure with minimized porosity, which improves mechanical properties [34,36]. The main LPBF process parameters are connected to laser energy density *LED* = P/v × h × t (J/mm^3^) [1,25].

In conclusion, optimizing LPBF parameters is crucial for controlling the microstructure and ensuring desirable properties in dental Co-Cr alloys, such as high density, wear resistance, biocompatibility, and mechanical properties. LPBF parameters, laser power, distance between laser beam paths, layer thickness, and scanning speed, are most often studied, while there are no detailed analyses of the influence of base plate preheating on the structure and properties. Therefore, this work is a follow-up to the article [1], in which the samples (5/17 runs (parameters combination) in total) were selected based on the maximum and minimum values of *KV*_a_, *R*_ms_, and one central point of the CCD design and were analyzed in detail, while this paper analyzes the results of all 17/17 runs (combinations of parameters) and their influence on properties.

## 2. Materials and Methods

Test samples were fabricated using EOS CoCr SP2 (EOS GmbH, Krailling, Germany) metal powder on an LPBF machine AconityMINI (Aconity 3D GmbH, Herzogenrath, Germany) utilizing open-access process parameters through the AconitySTUDIO user interface. Seventeen combinations of parameters for fabrication were determined based on previous research as part of the doctoral thesis [37] and follow-up to the article [1]. In Table 1, the coded and actual levels of numeric variables are shown, which were used for the determination of 17 combinations of parameters used for fabrication specimens and they are shown in Table 2. Other LPBF fabrication parameters, layer thickness (t = 30 μm), hatch spacing (h = 60 μm), and laser beam diameter (D = 60 μm), were constant. A total of 17 batches were LPBF fabricated for static tensile testing (*n* = 5), three-point bending testing (*n* = 5), and toughness testing (*n* = 5), resulting in a total of 255 samples.

**Figure 1:**
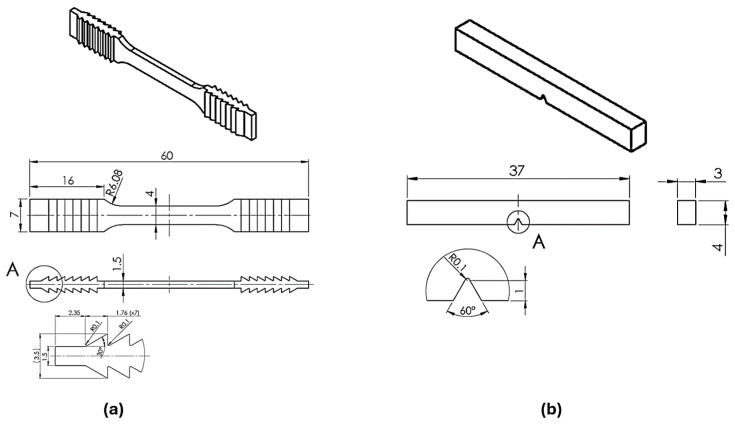
3D CAD model with dimensions of samples: (**a**) static tensile test and (**b**) toughness (V-notch).

Dimensions and form of test samples for static tensile testing and toughness are shown in Figure 1. The form and dimensions are non-standard due to the limited base plate radius Φ = 100 mm of the AconityMINI LPBF machine. The static tensile test (EN ISO 6892-1:2016 [38]) and impact fracture (EN ISO 148-1:2016 [39]) have been used as the requirements by the norms. Samples for three-point bending are defined by EN ISO 22674:2016 [40].

On a Shimadzu AGS-X device, a static tensile test was performed using an F_max_ 10 kN (Kyoto, Japan) with contact extensometer (*L*_0_ = 10 mm) (Figure 1a) to determine the elongation (ε) and yield strength *Rp*_0.2_ and three-point bending test to determine flexural strength (*R*_ms_). The toughness (*KV*_a_) test was performed (V notch samples, Figure 1b) on a Charpy impact machine (Karl Frank GmbH, Weinheim-Birkenau, Germany) with *L* = 21 mm. Hardness (HV1) was measured on a ZwickRoell ZHVμ-ST device (Indentec Ltd., West Midlands, UK) in cross- and longitudinal sections following EN ISO 6507-1:2018 [41].

**Figure 2:**
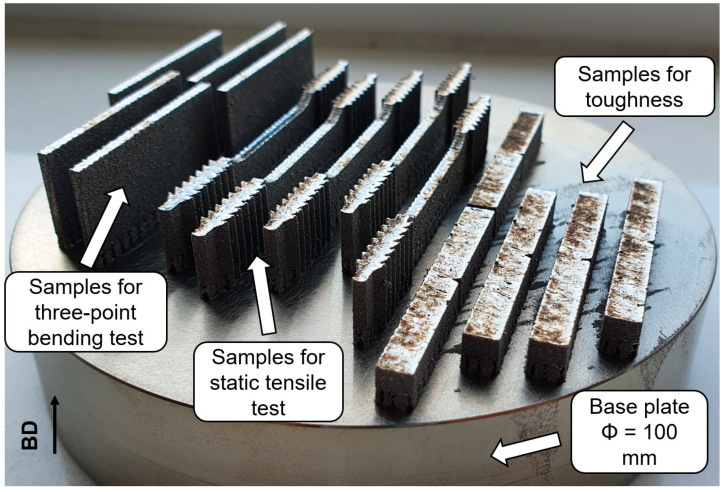
LPBF fabricated samples on the base plate.

Samples after LPBF fabricating are shown in Figure 2.

For the microstructure analysis, specimens were prepared with the standard metallographic procedure (electrochemical etching, 10 vol % oxalic acid, 12 V for 3 min). The microstructure was analyzed on an OLYMPUS GX51F-5 light microscope with a DP-25 CCD camera (Olympus Corporation, Shinjuku City, Tokyo, Japan), while the SEM TESCAN VEGA TS5136LS device with EDS (TESCAN, Brno, Czech Republic) was used for analysis.

The phase composition (detection of the γ_FCC_ and ε_HCP_ phases) was carried out using XRD (X-ray diffraction) analysis with BRUKER D8 DISCOVER (Bruker GmbH, Karlsruhe, Germany) device (Cu Kα copper anode (λ = 0.15406 nm)).

The conducted mechanical properties were analyzed using Design Expert^®^ ver. 11 (Stat-Ease, Inc.; Minneapolis, MN, USA), and the same mechanical properties between different groups (samples) were evaluated with one-way ANOVA and Scheffé post hoc test (α = 0.05).

## 3. Results

Toughness (*KV*_a_) and flexural strength (*R*_ms_) are crucial mechanical properties for dental application because the lower jaw undergoes various forces: compression, tension, and shear during both functional and non-functional movements, including contact with opposing teeth [1,42]. The Co-Cr material used in dental prostheses must withstand these forces, especially in bridge constructions that replace one or more missing teeth [43]. In metal–ceramic restorations, the Co-Cr alloy forms the structural core, providing the necessary flexural strength and toughness to support the fragile ceramic overlay [1,44,45]. Because of those features, a detailed analysis of *KV*_a_ and *R*_ms_ was carried out, response surfaces were designed, and mathematical models were defined that describe them and serve to predict individual properties. *Rp*_0.2_ and *ε* were tested to prove that all manufactured samples meet the EN ISO 22674 standard for dental applications [40].

Table 3 shows all the results (mean values with standard deviations, *n* = 5 per group) of mechanical tests for a total of 255 samples.

Table 4 shows all the results of hardness HV1 (mean values with standard deviations, *n* = 10 per group) in the cross-section and longitudinal section.

**Figure 3:**
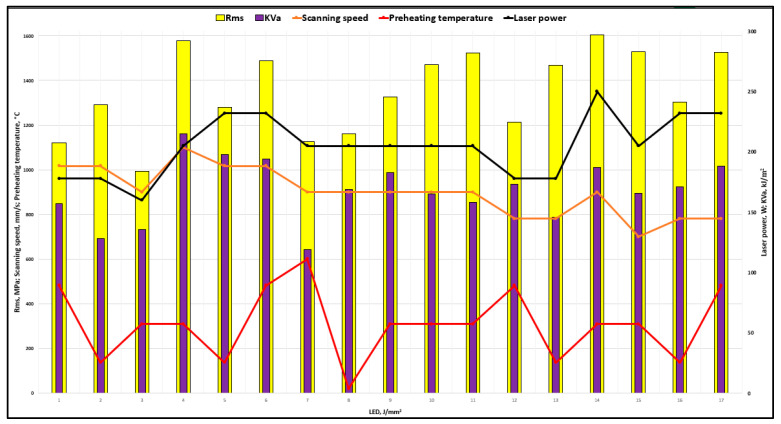
Influence of LPBF fabrication parameters and laser energy density on toughness and flexural strength.

**Figure 4:**
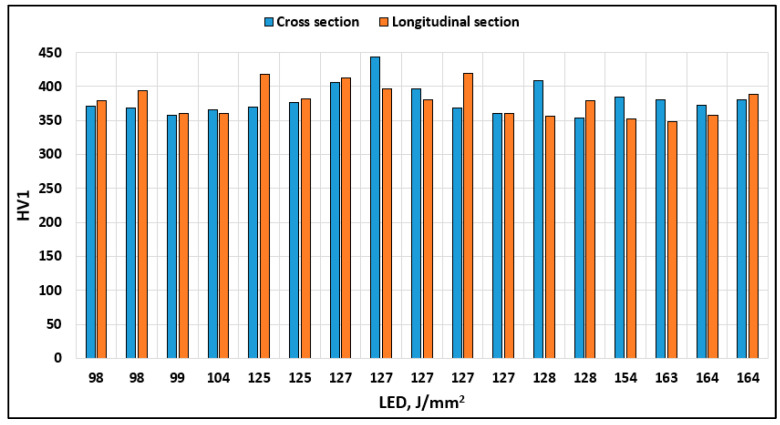
Influence of laser energy density on hardness HV1.

Mechanical properties (important for dental application, *R*_ms_, and *KV*_a_) obtained by mechanical tests from Table 3 are shown graphically in Figure 3, while Figure 4 shows hardness HV1 in cross- and longitudinal sections in dependence of LED.

### 3.1. Microstructure and XRD Analysis

**Figure 5:**
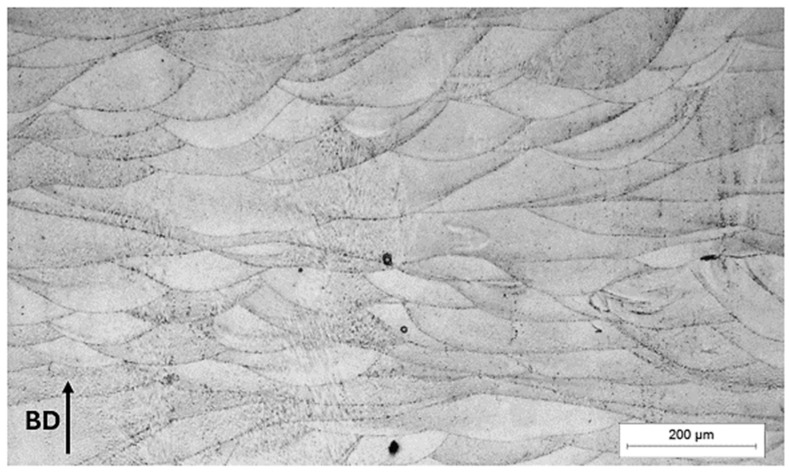
Sample 9, LM: microstructure, etched.

All samples (*n* = 17 batches) of the experiment in the cross-section have a dendritic microstructure with recognizable solidified arched shapes melt pools (Figure 5). The analysis of the microstructure in the cross-section concluded that the microstructure of all test samples does not differ.

**Figure 6:**
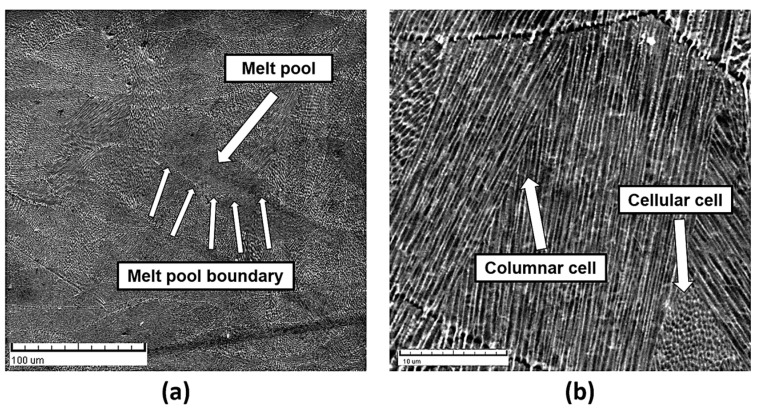
Sample 5, SEM: (**a**) melt pool with boundary and (**b**) cellular–dendritic microstructure.

Figure 6 shows the microstructure analyzed by SEM, and it is evident that it is a recognizable cross-section microstructure of arched shape melt pools with boundary (Figure 6a), while Figure 6b shows the fine-grained cellular–dendritic microstructure consisting of cellular and columnar cells.

**Figure 7:**
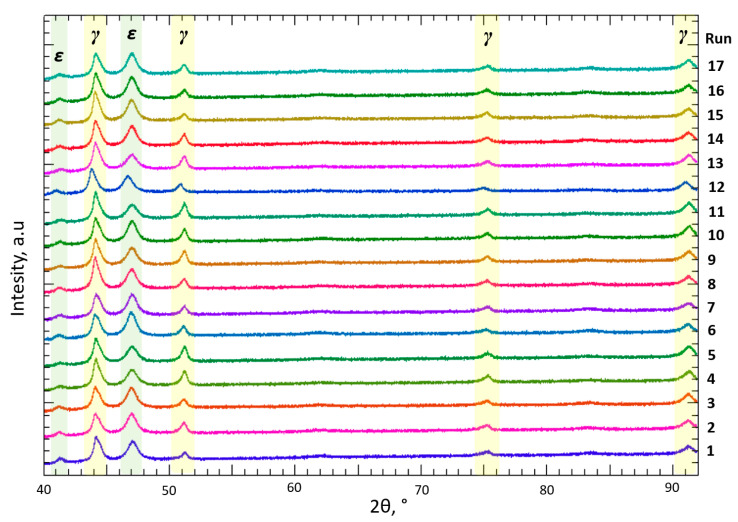
XRD analysis of runs. Each color represents an XRD graph, a total of 17 graphs (colors) for 17 runs.

Two microstructural phases γ_FCC_ and ε_HCP_ were detected by XRD analysis of all samples of 17 parameters combination shown in Figure 7. The spectra were indexed [25,46] and prove the existence of two phases, γ_FCC_ (Co-fcc, ICDD:15-806) and ε_HCP_ (Co-hcp, ICDD:5-727), in all Co-Cr samples tested (17 runs), regardless of the used LPBF parameters. The diffractograms in Figure 7 show the presence of γ_FCC_ and ε_HCP_ phases at the same angles but with varying intensities.

### 3.2. Model Result and Statistical Analysis

The mechanical properties obtained from experimental tests (Table 3) were statistically analyzed, and models were developed to quantify the impact of LPBF production parameters on the measured size (mechanical properties) using the Design Expert^®^ ver. 11 (Stat-Ease, Inc.; USA). To define the optimal production parameters, the response surface methodology (RSM) was applied, which mathematically connects the experimental system with the theoretical design via the objective function [24,47,48]. ANOVA was used to determine the significant parameters of LPBF processing (Table 5 and Table 6) [49]. The reliability of the model was determined using the F-test (*p*-value) and using the coefficients that describe the model [47,49].

Using the CCD model, the proposed experiments were conducted, and the results for the evaluated responses are shown in Table 3. From the ANOVA table generated by RSM, a cubic polynomial regression model was developed to assess the impact of the independent parameters on both responses (*R*_ms_ and *KV*_a_). The final equations for both responses, expressed in terms of actual factors, are as follows:

(1) $$\begin{array}{l} R_{ms}=78458.271-472.00883\;A-149.10379\;B-2.63918\;C+0.834559\;AB\\ \;+0.023191\;AC+0.517561\;A^{2}+0.066833\;B^{2}-0.003449\;C^{2}\\ \;-0.000649\;A^{2}B-{0.000311\;AB}^{2} \end{array}$$

(2) $$\begin{array}{l} KV_{a}=4791.58299-39.95391\;A-8.77176\;B+8.31107\;C+0.073386\;AB\\ \;-0.079177\;AC+0.094105A^{2}+0.000612\;B^{2}-0.000262\;C^{2}\\ \;-0.000173\;A^{2}B+0.000190\;A^{2}C \end{array}$$

ANOVA analysis of experimental data is shown in Table 5 (*R*_ms_) and Table 6 (*KV*_a_). The significant *F*-values (13.55, 11.22) and *p*-values < 0.05 (for both responses; 0.0023, 0.0039) have confirmed the acceptance of the developed polynomial cubic model equation. The values of R^2^ (0.9576, 0.9492), R^2^_adj_ (0.8869, 0.8646), and R^2^_pred_ (0.8496, 0.5776) confirmed that the cubic polynomial model indicates the interconnection for responses. The adequate precisions are 12.1237 and 12.6324 (>4), which also proves the developed cubic polynomial models.

For optimization, the condition of the maximum value of *R*_ms_ and *KV*_a_ was defined while limiting the input parameters in the tested range of values. The suggested optimal LPBF input parameters to achieve the maximum values are as follows:

- *KV*_a_: P = 234 W, v = 1078 mm/s and *ϑ*_p_ = 380 °C
- *R*_ms_: P = 246 W, v = 828 mm/s and *ϑ*_p_ = 331 °C.

### 3.3. Response Surface Analysis

**Figure 8:**
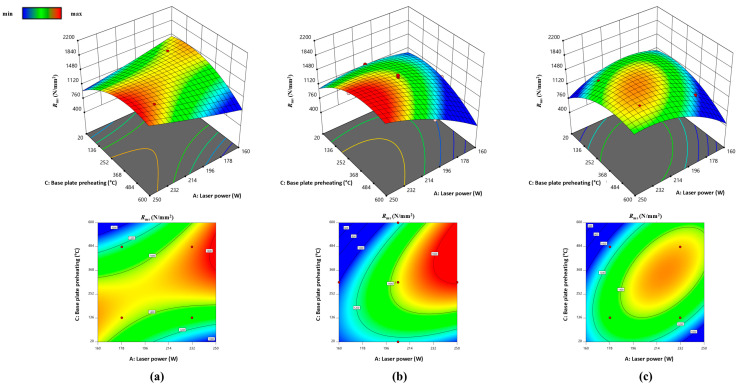
RSM: combined interaction of laser power and base plate preheating on *R*_ms_ at different scanning speeds *v*: (**a**) 780 mm/s, (**b**) 900 mm/s, and (**c**) 1020 mm/s.

**Figure 9:**
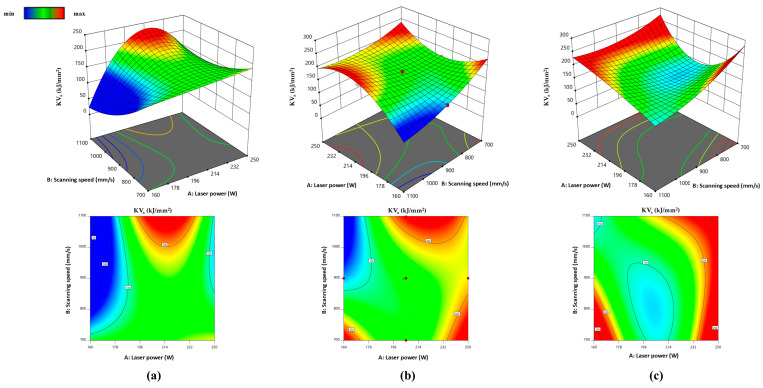
RSM: combined interaction of laser power and scanning speed on *KV*_a_ at different base plate preheating *ϑ*_p_: (**a**) 140 °C, (**b**) 310 °C, and (**c**) 480 °C.

Figure 8 and Figure 9 show interaction (laser power and base plate preheating for *R*_ms_ and laser power and scanning speed for *KV*_a_) at three different levels (scanning speed and base plate preheating) while other variables (t = 30 μm, h = 60 μm, and D = 60 μm) were constant.

According to the response surfaces in Figure 8 and Figure 9 and the results from Table 3 and Figure 3, it can be concluded that increasing the laser power above 200 W generally leads to higher toughness values, particularly when combined with high scanning speeds (v > 1000 mm/s) or when base plate preheating temperatures are lower (*ϑ*_p_ = 140 °C). This suggests that higher energy input promotes better fusion, leading to enhanced toughness. Toughness values are sensitive to the interaction between scanning speed and laser power. At higher scanning speeds, toughness improves with increasing laser power, while at lower speeds, optimal toughness is achieved with moderate laser power. When the base plate is preheated to 310 °C, the optimal toughness is achieved at higher laser powers (*P* > 230 W) and scanning speeds around 900 mm/s. At higher preheating temperatures (*ϑ*_p_ = 480 °C), toughness improves at lower laser powers, indicating a balance between energy input and controlled solidification.

Flexural strength increases with laser power above *P* > 205 W, reaching maximum values when the base plate is preheated to 310 °C. This is because higher laser power ensures better melting and reduces porosity, enhancing strength. Base plate preheating to 310–480 °C improves *R*_ms_, particularly when combined with moderate to high laser powers. Preheating reduces thermal gradients and promotes a finer grain structure, resulting in better mechanical properties.

Toughness (*KV*_a_) and flexural strength (R_ms_) are influenced by the interaction of laser power, scanning speed, and base plate preheating temperature. Higher laser power and optimal preheating temperature (*ϑ*_p_ = 310 °C) are critical for achieving the optimal mechanical properties.

### 3.4. Validation of the Optimal LPBF Parameters to Obtain the Maximum KV_a_ Value

The optimal LPBF parameters for achieving the maximum *KV*_a_ value were additionally confirmed by conducting mechanical tests on samples produced with LPBF-selected optimal parameters. The mean values of the mechanical tests (*n* = 5) were compared with the results obtained by predicting the model (Equation (2)). The values of *KV*_a_ predicted by the model and obtained by mechanical tests (Table 7) do not differ significantly, and the calculated difference between them is less than 5 %, which successfully validates the optimal parameters.

## 4. Discussion

An extensive analysis was conducted utilizing light and electron microscopy to determine the microstructural characteristics of test samples produced with the LPBF process. Results indicated a consistent cellular–dendritic microstructure across all 17 experimental runs, with clearly defined boundaries between the solidified regions caused by the laser beam passage, irrespective of the LPBF process parameters. Additionally, microstructure analysis revealed consistent findings, with no discernible variations associated with the input parameters.

XRD analysis further revealed the presence of the crystallographic phases *γ*_FCC_ and *ε*_HCP_ across all test samples, indicating minimal influence of the production parameters on the formation of microstructural phases. Notably, a detailed analysis of the intensity of individual phases demonstrated a correlation between intensity and the proportion of the respective microstructural phase.

Mechanical testing confirmed compliance with dental standard HRN EN ISO 22674:2016 for all test samples in terms of *Rp*_0.2_ (>500 N/mm^2^) and ε (>2%) values, meeting the minimum criteria for use in the production of dental prosthetics of type 5 [40]. Moreover, hardness measurements indicated negligible impact of the input LPBF parameters on HV1 values across both sections and test samples (Figure 4 and Table 4).

Using a CCD design and statistical processing, mathematical models and response surfaces were defined for mechanical properties, such as toughness (*KV*_a_) and flexural strength (*R*_ms_). The analysis revealed a significant influence of specific input LPBF parameters on individual mechanical properties, parameters *P*, *ϑ*_p_, and v significantly impacting *KV*_a_ values (*p* < 0.05), and laser power *P* significantly affecting *R*_ms_ values (*p* < 0.05).

The results indicated that higher laser power (*P* > 205 W) and intermediate scanning speeds (*v* > 1000 mm/s) led to improved toughness values *KV*_a_, especially when the base plate preheating temperature was set at *ϑ*_p_ = 310 °C. This indicates that high energy input results in better material fusion, reducing defects and enhancing toughness.

Flexural strength *R*_ms_ improved with increased laser power and moderate scanning speeds. The optimal mechanical properties were achieved with higher laser power (*P* > 230 W) and base plate preheating temperatures *ϑ*_p_ = 310 °C. Preheating reduces thermal gradients, promoting a uniform microstructure and minimizing residual stress.

By comparing the mechanical properties of LPBF-produced dental Co-Cr alloys from the literature with the mechanical tests obtained in this research, they are comparable, but it should be noted that the alloys from the literature were subsequently heat-treated after the LPBF procedure, while the test samples used in the work were not subjected to subsequent heat treatments [5,25,50,51,52,53].

It should also be noted that dental Co-Cr alloys produced by different production methods do not have the same chemical composition because no alloy with the same characteristics is available for three different production techniques [17]. The properties of dental Co-Cr alloys can be influenced not only by the main elements but also by alloying elements (Mo, W, C, N) [54,55]. For this reason, direct comparison methods cannot be connected only with different production technologies, but all subsequent types of processing should also be considered, which also represents a limitation of the conducted comparison.

## 5. Conclusions

- The highest values of toughness (*KV*_a_), flexural strength (*R*_ms_), yield strength (*Rp*_0.2_), and elongation (*ε*) were obtained with LED > 125 J/mm^2^ using a base plate preheating temperature range *ϑ*_p_ = 310 °C–480 °C and laser power *P* > 205 W, regardless of scanning speed values (*v*).
  Base plate preheating temperature (*ϑ*_p_) and laser power (*P*) significantly affect the mechanical properties of the fabricated samples. Increasing the laser power at higher preheating temperatures ensured an LED value sufficient to fully melt layer on layer, producing a uniform microstructure with reduced porosity and finer grain size.
- Using the input parameters of SLM from test conditions 3, 7, 9, and 10, samples were produced that possess combinations of mechanical properties similar to or higher in comparison with those obtained through conventional manufacturing methods.
- Optimal LPBF parameters for achieving maximum values of individual mechanical properties were determined using defined models. The optimal parameters for toughness (*KV*_a_) are *P* = 234 W, *v* = 1078 mm/s, *ϑ*_p_ = 380 °C. The optimal parameters for flexural strength (*R*_ms_) are *P* = 246 W, *v* = 828 mm/s, and *ϑ*_p_ = 331 °C.
- Validation of the optimal LPBF parameters for achieving maximum toughness (*KV*_a_) was conducted. It was found that the obtained mathematical model predicts toughness (*KV*_a_) values with a difference of less than 5% compared to values obtained from mechanical testing.
- Mechanical testing and material characterization have demonstrated that all 17 test conditions meet the standard HRN EN ISO 22674:2016 for the use of materials in dental restorations, regardless of the input parameters of the LPBF used in this study.

Possible directions of future research are suggested, which would include heat treatment of samples after the LPBF process to carry out a more accurate comparison with other production technologies.

## Figures and Tables

**Table 1:** Coded and actual levels of numeric variables for CCD.

Level of Variables	Symbol: Variables, Units		
	A: *P,* W	B: *v*, mm/s	C: *ϑ_p_*, °C
−1.682	160	700	20
−1	178	781	137
0	205	900	310
1	231	1018	482
1.682	250	1100	600

**Table 2:** Design experiments for LPBF fabrication.

Run	A: Laser Power, W	B: Scanning Speed, mm/s	C: Base Plate Preheating, °C
1	231	1018	137
2	231	781	137
3	205	1100	310
4	178	781	482
5	205	900	600
6	178	781	137
7	178	1018	137
8	205	900	20
9	231	1018	482
10	205	900	310
11	178	1018	482
12	205	700	310
13	231	781	482
14	205	900	310
15	160	900	310
16	205	900	310
17	250	900	310

**Table 3:** Conducted mechanical properties: mean values with standard deviations.

Run	LED	*KV*_a_	*ε*	*R*_p0.2_	*R*_ms_
	J/mm^2^	kJ/m^2^	%	N/mm^2^	N/mm^2^
11	98	157 ± 12 ^b^	6.5 ± 1 ^a^	783 ± 41 ^b^	1122 ± 78 ^b^
7	98	128 ± 8 ^a^	11.2 ± 0.9 ^b^	773 ± 37 ^b^	1292 ± 66 ^c^
15	99	136 ± 23 ^a,b^	7.6 ± 1.3 ^a^	653 ± 30 ^a^	994 ± 43 ^a^
3	104	215 ± 8 ^d^	15.9 ± 0.2 ^c^	884 ± 13 ^c^	1578 ± 67 ^e^
1	125	198 ± 9 ^c^	9.2 ± 1.9 ^a,b^	742 ± 54 ^a,b^	1281 ± 22 ^c^
9	125	194 ± 7 ^c^	9 ± 0.3 ^b^	981 ± 38 ^d^	1488 ± 29 ^d^
5	127	119 ± 7 ^a^	9.1 ± 0.8 ^b^	745 ± 92 ^a,b^	1126 ± 24 ^b^
8	127	169 ± 2 ^b^	10.8 ± 1.8 ^b^	919 ± 92 ^b,c,d^	1161 ± 37 ^b^
16	127	183 ± 4 ^c^	7.3 ± 0.9 ^a^	748 ± 64 ^a,b^	1327 ± 23 ^c^
14	127	165 ± 20 ^b,c^	6.8 ± 1.4 ^a^	719 ± 33 ^a,b^	1472 ± 33 ^d^
10	127	158 ± 12 ^b^	12.2 ± 0.6 ^b^	822 ± 70 ^b,c^	1523 ± 46 ^d,e^
4	128	173 ± 13 ^b^	6.5 ± 0.3 ^a^	873 ± 19 ^c^	1212 ± 41 ^c^
6	128	146 ± 10 ^a,b^	10.4 ± 1 ^b^	857 ± 45 ^b,c^	1468 ± 11 ^d^
17	154	187 ± 6 ^c^	8.3 ± 2 ^a,b^	694 ± 76 ^a,b^	1603 ± 23 ^e^
12	163	166 ± 18 ^b,c^	6.2 ± 0.8 ^a^	962 ± 28 ^d^	1530 ± 70 ^d,e^
2	164	171 ± 13 ^b,c^	7.7 ± 0.5 ^a^	845 ± 36 ^b,c^	1304 ± 12 ^c^
13	164	188 ± 7 ^c^	6.7 ± 0.7 ^a^	924 ± 8 ^d^	1527 ± 40 ^d,e^

Different superscript letters in a column indicate statistically significant differences (*p* < 0.05).

**Table 4:** Mean values and standard deviations of hardness.

Run	LED	Hardnes, HV1	
	J/mm^2^	Cross-Section	Longitudinal Section
11	98	371 ± 8 ^a,b^	379 ± 12 ^b^
7	98	369 ± 13 ^a,b^	394 ± 20 ^b,c^
15	99	358 ± 6 ^a^	361 ± 7 ^a,b^
3	104	366 ± 10 ^a,b^	361 ± 12 ^a,b^
1	125	370 ± 10 ^a,b^	418 ± 12 ^c^
9	125	376 ± 16 ^a,b^	382 ± 12 ^b^
5	127	406 ± 8 ^c^	413 ± 7 ^c^
8	127	443 ± 15 ^d^	396 ± 6 ^c^
16	127	397 ± 11 ^b,c^	381 ± 16 ^b,c^
14	127	369 ± 18 ^a,b^	420 ± 12 ^c^
10	127	360 ± 6 ^a^	360 ± 7 ^a,b^
4	128	408 ± 6 ^c^	357 ± 3 ^a^
6	128	354 ± 6 ^a^	379 ± 11 ^b^
17	154	385 ± 10 ^b^	353 ± 3 ^a^
12	163	380 ± 3 ^b^	348 ± 9 ^a^
2	164	373 ± 22 ^a,b^	358 ± 3 ^a^
13	164	381 ± 8 ^b^	389 ± 8 ^b, c^

Different superscript letters in a column indicate statistically significant differences (*p* < 0.05).

**Table 5:** ANOVA and statistical values for *R*_ms_ model.

Source	Sum of Squares	df	Mean Square	*F*-Value	*p*-Value	
Model	5283 × 10^5^	10	52,833.83	13.55	0.0023	Significant
A	1.854 × 10^5^	1	1.854 × 10^5^	47.55	0.0005	
B	1152.00	1	1152.00	0.2954	0.6064	
C	220.40	1	220.40	0.0565	0.8200	
AB	5202.00	1	5202.00	1.33	0.2920	
AC	91,592.00	1	91,592.00	23.49	0.0029	
A^2^	25,699.34	1	25,699.34	6.59	0.0425	
B^2^	20,446.35	1	20,446.35	5.24	0.0620	
C^2^	1.185 × 10^5^	1	1.185 × 10^5^	30.40	0.0015	
A^2^B	10,122.80	1	10,122.80	2.60	0.1583	
AB^2^	45,989.10	1	45,989.10	11.79	0.0139	
Residual	23,397.79	6	3899.63			
Lack of fit	2717.13	4	679.28	0.0657	0.9865	Not significant
Pure error	20,680.67	2	10,340.33			
Cor total	5.51 × 10^5^	16				

R^2^ = 0.9576, R^2^_adj_ = 0.8869, R^2^_pred_ = 0.8496, Adequate Precision = 12.1237. *p* < 0.05 is significant.

**Table 6:** ANOVA and statistical values for the *KV*_a_ model.

Source	Sum of Squares	df	Mean Square	*F*-Value	*p*-Value	
Model	10,012.95	10	1001.29	11.22	0.0039	Significant
A	3967.42	1	3967.42	44.45	0.0006	
B	1200.50	1	1200.50	13.45	0.0105	
C	1250.00	1	1250.00	14.00	0.0096	
AB	561.13	1	561.13	6.29	0.0461	
AC	231.13	1	231.13	2.59	0.1587	
A^2^	28.56	1	28.56	0.32	0.5921	
B^2^	845.73	1	845.73	9.48	0.0217	
C^2^	682.16	1	682.16	7.64	0.0327	
A^2^B	715.36	1	715.36	8.01	0.0299	
A^2^C	1828.45	1	1828.45	20.49	0.0040	
Residual	535.52	6	89.25			
Lack of fit	202.86	4	50.71	0.3049	0.8565	Not significant
Pure error	332.67	2	166.33			
Cor total	10,548.47	16				

R^2^ = 0.9492, R^2^_adj_ = 0.8646, R^2^_pred_ = 0.5776, Adequate Precision = 12.6324. *p* < 0.05 is significant.

**Table 7:** Validation of optimal LPBF parameters for *KV*_a_.

Optimal LPBF Parameters			*KV*_a_, kJ/m^2^	
*P*, W	*v*, mm/s	*ϑ*_p_, °C	Predicted	Experimental
234	1078	380	215	206 ± 5

## Data Availability

The data presented in this study are openly available in [Repository of Faculty of Mechanical Engineering and Naval Architecture University of Zagreb] at https://repozitorij.fsb.unizg.hr/islandora/object/fsb:9493 (accessed on 10 September 2024).
